# How one nutrient controls cell size

**DOI:** 10.7554/eLife.109482

**Published:** 2025-11-19

**Authors:** Angela Montero, Lydia WS Finley

**Affiliations:** 1 Memorial Sloan Kettering Cancer Center New York United States

**Keywords:** pyruvate metabolism, redox state, cell growth, hepatocytes, translation, genetics, *D. melanogaster*, Human

## Abstract

The metabolic fate of a nutrient called pyruvate determines how big cells become.

**Related research article** Toshniwal AG, Lam G, Bott AJ, Cluntun AA, Skabelund R, Nam HJ, Wisidagama DR, Thummel CS, Rutter J. 2024. The fate of pyruvate dictates cell growth by modulating cellular redox potential. eLife **13**:RP103705. doi: 10.7554/eLife.103705.

Cells are the basic unit of life and come in many different sizes and shapes. In animals, cell size is exquisitely matched to cell function: the smallest cells (such as red blood cells and sperm) shed organelles to focus on carrying their unique cargos, whereas the largest cells (such as eggs and neurons) grow in volume or length to stockpile nutrients or traverse long distances.

Cells can also grow or shrink in response to environmental cues. For example, naïve T cells increase in size before undergoing a burst of proliferation, while liver and fat cells enlarge or contract in response to nutrient availability. In these cases, size changes are directed by kinases (such as PI3K/Akt and mTORC1) and transcription factors (such as c-Myc) that reorganize cellular metabolic networks to prioritize pathways that build biomass over catabolic pathways that consume nutrients (Liu et al., 2022; Lloyd, 2013).

Despite the central role of metabolism in enabling cell growth, metabolites have largely been viewed as effectors rather than drivers of cell size. Now, in eLife, Jared Rutter and colleagues at the University of Utah – including Ashish Toshniwal as first author – report the results of experiments that overturn this hierarchy (Toshniwal et al., 2024). They show that the metabolic fate of one specific nutrient called pyruvate is the dominant factor controlling the size of fat body cells in *Drosophila* and liver cells in mammals.

Pyruvate, produced through the breakdown of sugars, sits at central node of metabolism. Pyruvate can either be burned to fuel energy production or converted to building blocks that enable cell growth. The fate of pyruvate, therefore, has significant implications for metabolic pathways. For instance, increasing pyruvate import into mitochondria reduces proliferation in various progenitor cells (Schell et al., 2017; Bensard et al., 2020). Conversely, in the adult heart, where cells no longer proliferate, reducing mitochondrial pyruvate import leads to increased cell size (Cluntun et al., 2021). These observations raise the possibility that pyruvate – or the effects of its metabolism – may also regulate cell size programs.

To test this, Toshniwal et al. examined fat body cells of fruit flies during the third instar stage of larval development, when cells undergo orchestrated increases in size. They found that as cell size increased, the levels of a molecule that imports pyruvate into mitochondria, the mitochondrial pyruvate carrier (MPC), decreased. This downregulation was essential for normal cell growth: experimentally increasing MPC expression stopped cells from enlarging, whereas further reducing MPC led to cells becoming larger.

Surprisingly, MPC-regulated control of size did not operate through canonical growth pathways. In fact, MPC expression reduced cell size despite activation of mTORC1 and c-Myc. Even driving hyperactive PI3K/Akt, mTORC1 and c-Myc – each normally sufficient to increase cell size – failed to do so in cells that overexpressed MPC. These results demonstrate that importing pyruvate into mitochondria overrides conventional signaling pathways to control cell size.

How does mitochondrial pyruvate import control cell size? When pyruvate enters mitochondria, several metabolic consequences converge to decrease cell size (Figure 1). First, pyruvate is no longer available for reduction to lactate in the cytosol, a reaction that regenerates NAD^+^ (the oxidized form of nicotinamide adenine dinucleotide) from NADH (the reduced form). Accordingly, MPC-overexpressing cells accumulate NADH at the expense of NAD^+^. Second, mitochondrial pyruvate generates oxaloacetate, a key substrate for glucose production. Together, oxaloacetate production and NADH accumulation activate glucose production in MPC-overexpressing cells. Blocking the production of glucose reversed this size reduction, indicating that persistent glucose production contributes to a smaller cell size.

**Figure 1.**
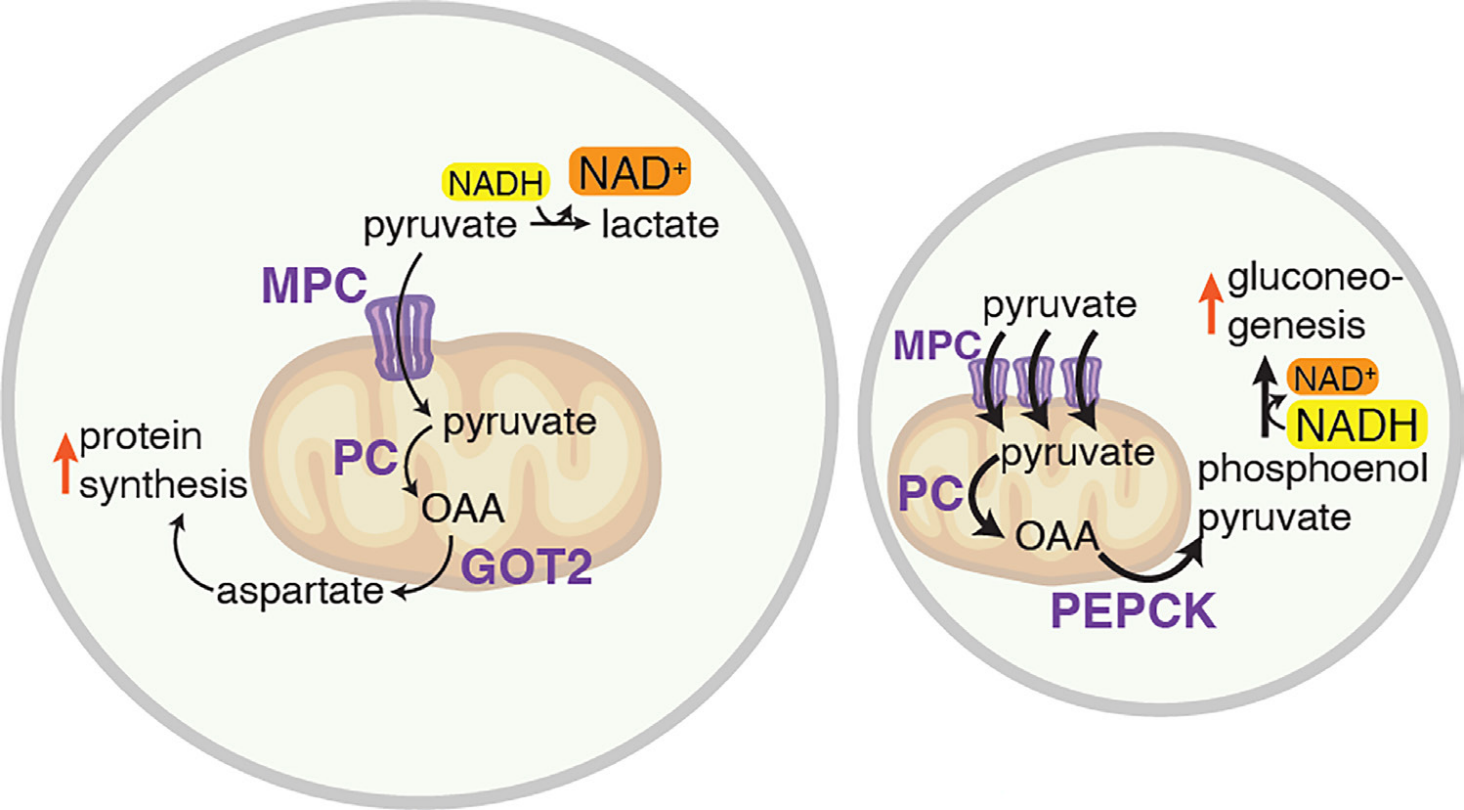
How pyruvate entry into the mitochondria determines cell size. Left: When the mitochondrial pyruvate carrier (MPC) is expressed at low levels, pyruvate in the cytosol (yellow) can either enter mitochondria or be converted to lactate by an enzyme called LDH. Limited pyruvate entry into mitochondria enables LDH to maintain high levels of NAD^+^ in the cytosol. In mitochondria, pyruvate can be converted to oxaloacetate (OAA) by an enzyme called pyruvate carboxylase (PC). OOA is then converted to aspartate by an enzyme called GOT2. This production of aspartate supports protein synthesis, thereby increasing cell size. Right: When MPC is overexpressed, pyruvate entry into mitochondria increases, disrupting the balance of NAD^+^ and NADH in the cytosol. In mitochondria, pyruvate can be converted to oxaloacetate, which is then converted to phosphoenolpyruvate by an enzyme called PEPCK. The high levels of phosphoenolpyruvate (rather than aspartate) in the cytosol lead to an increase in glucose production (gluconeogenesis) and a reduction in protein synthesis, resulting in a smaller cell size. LDH: lactate dehydrogenase; NAD: nicotinamide adenine dinucleotide; GOT2: Glutamic-oxaloacetic transaminase; PEPCK: phosphoenolpyruvate carboxykinase.

MPC-expressing cells were smaller because of reduced protein synthesis. When oxaloacetate is used to produce glucose, it can no longer make amino acids such as aspartate, which is required for protein and nucleotide biosynthesis. Restoring amino acid availability rescued cell size in MPC-overexpressing cells. These findings suggest that glucose production and protein synthesis compete for oxaloacetate, and that the cellular redox state determines which pathway predominates. Consistently, increasing NAD^+^ at the expense of NADH counteracted the size-reducing effect of MPC expression. Whether NAD^+^/NADH affects protein synthesis through enzymatic control, signaling, or other mechanisms remains to be determined.

Together, these results indicate that the metabolic fate of pyruvate triggers a cascade of events that determine cell size. During typical development, cells undergo tightly controlled changes in cell size. To date, these processes are thought to be under the control of extracellular signaling cues that drive transcriptional programs that define phenotypic traits like cell shape, size and protein composition.

However, development is often accompanied by substantial shifts in metabolic state, particularly in how pyruvate is processed (Cliff et al., 2017; Jackson et al., 2025; Jackson and Finley, 2024). The work of Toshniwal et al. demonstrates that these changes are not only mere consequences of developmental programs but can also act as key determinants of cell size and physiology.

The alternative fates of pyruvate – be it energy generation, amino acid synthesis or glucose production – will determine the capacity of cells to engage different metabolic processes. In the liver and fat cells, changing how pyruvate is used may therefore affect whether cells burn or store nutrients in response to organismal demands. More broadly, maintaining an appropriate size is critical for proper cellular function. This work raises the possibility that the fate of pyruvate – and the resulting downstream metabolic trade-offs – ensures that cell size is matched to metabolic capacity in various contexts.
